# Compositional Analysis of Non-Polar and Polar Metabolites in 14 Soybeans Using Spectroscopy and Chromatography Tools

**DOI:** 10.3390/foods8110557

**Published:** 2019-11-07

**Authors:** Raghavendhar R. Kotha, Savithiry Natarajan, Dechun Wang, Devanand L. Luthria

**Affiliations:** 1 USDA-ARS, Beltsville Human Nutrition Research Center, Beltsville, MD 20705, USA; raghukotha123@gmail.com; 2 USDA-ARS, Soybean Genomics and Improvement Laboratory, Beltsville, MD 20705, USA; savi.natarajan@usda.gov; 3 Department of Plant, Soil, and Microbial Sciences, Michigan State University, East Lansing, MI 48824, USA; wangdech@msu.edu

**Keywords:** soybeans, multivariate analysis, non-polar and polar metabolites, ion and gas chromatographic separations, correlation studies

## Abstract

There has been significant interest in soybean oil, fatty acid, and sugar composition to develop new value-added soybean products. Thus, compositional analysis is critical for developing value-added soybeans. In the present study, we showed simple screening tools (near infrared spectroscopy (NIR) and high-performance thin layer chromatography (HPTLC)) coupled with multivariate analysis for the sample classification of 14 soybeans as a proof-of-concept. We further determined major non-polar and polar metabolites responsible for differences between different soybeans using gas and ion chromatography. These differences in soybean profiles were attributed to lower levels of total oil content in wild soybeans (~9%) versus cultivated soybeans (16%–22%). In addition, higher levels of linolenic acid (~17%) and stachyose (~53%) were determined in wild type, whereas higher levels of oleic acid (~19%) and sucrose (~59%) were detected in cultivated soybeans. Interestingly, one cultivated soybean had a desirable sugar profile with a high amount of sucrose (86%) and a low abundance of stachyose (9%). The correlation studies showed a positive correlation between oil and soluble sugars (*R*^2^ = 0.80) and negative correlations between methyl linolenate and soluble sugars (*R*^2^ = −0.79), oil (*R*^2^ = −0.94), and methyl oleate (*R*^2^ = −0.94) content. Both polar and non-polar metabolites showed significant differences in wild and cultivated soybeans.

## 1. Introduction

Soybeans are produced and consumed globally. About 360 million metric tons of soybean have been produced across the globe in 2018 [1]. The United States is the world’s leading soybean producer, accounting for approximately over one-third (worth of about $40,000 million dollars) of total global production [1]. Soybean has been an important source of food and animal feed owing to its protein, oil, and carbohydrate content [2]. Soybean-based foods and food products account for 30% and 69% of dietary oil and protein, respectively [3]. Soybean seeds generally contain about 40% protein, 35% carbohydrate, 20% oil, and 5% ash and other phytochemicals [2,4]. Soybeans comprise about 90% of the United States’ oilseeds production. In addition, soybean seeds contain other active ingredients such as isoflavones, saponins, and lecithins.

In recent years, there has been significant interest in the fatty acid composition of the oil owing to its nutritional and health significance [5]. The fatty acid composition of conventional soybean oil primarily comprises around 80%–85% unsaturated fatty acids (~7%–10% linolenic acid (18:3); ~50% linoleic acid (18:2); ~20% oleic acid (18:1); and between 10% and 20% saturated fatty acids, namely palmitic acid (16:0) and stearic acid (18:0) [5,6]. The lower concentration of polyunsaturated fatty acids (18:3) is always desirable as it reduces the shelf-life owing to oxidation of polyunsaturated fatty acids [7]. Researchers use breeding/transgenic/mutagenesis approaches to silence/remove the genes associated with the biosynthesis of polyunsaturated fatty acids to modify the fatty acids profile [6,8]. Furthermore, it has been shown that a higher concentration of oleic acid in oils results in lowering the concentration of low-density lipoprotein (LDL)-cholesterol without decreasing the high-density lipoproteins (HDL) cholesterol, thereby improving the nutritional quality of soybeans [9].

It is well known that sugars serve as a primary precursor for fat biosynthesis in plants [10], hence the understanding of sugars in soybeans may provide a better understanding of fat content. Hymowitz et al. showed a positive correlation between sugar and oil content in sixty selected soybean samples [11]. Furthermore, the sugar profiles have a direct impact on the soy food quality, digestibility, and nutritive value [2,4]. The commonly reported soluble carbohydrates in soybean seeds are monosaccharides (glucose, galactose, and fructose), disaccharides (sucrose), and oligosaccharides (raffinose and stachyose) [12,13]. Soybean seed contains about 30%–40% of total carbohydrates as soluble sugars [2,4], which prominently include sucrose (40%–70%); raffinose (5%–15%); stachyose (10%–40%) [2,13,14]; and low quantities (<5%) of glucose, fructose, and galactose. Glucose, fructose, and sucrose are easily digested and these sugars also provide desirable taste and flavor to many soy foods such as soymilk, tofu, natto, bean sprouts, and edamame [2,4]. Meanwhile, oligomeric sugars such as raffinose and stachyose are not easily digested and may also result in flatulence and diarrhea [4]. The insoluble polysaccharides found in soybean are pectin, cellulose, hemicellulose, and starch [12]. It is well documented in the literature that carbohydrates levels in soybean seed are significantly altered by growing conditions [15], genotype [16], maturity, and their interactions [17].

Classical targeted analysis often involves separation and extraction of analytes [18]. This approach often requires extraction, partial purification, and analysis of the targeted analyte/analytes/class of compounds of interest [18]. Partial purification is achieved by solvent extractions, sep-pack cartridge purification, size exclusion chromatography, precipitation, and other techniques [19,20,21]. Several chromatographic methods including gas chromatography (GC), high-performance liquid chromatography (HPLC), ion chromatography (IC), gel permeation chromatography (GPC), supercritical fluid chromatography (SFC), and affinity chromatography coupled with multiple detections methods (mass spectrometry (MS), tendem mass spectrometry (MS)^n^, UV/visible (UV/vis), flame ionization (FID), and electrochemical (ECD)) have been used to quantify metabolites from various matrices [13,22,23,24,25]. These techniques are expensive and time consuming, thus limiting the practical commercial application in the food and agriculture industry. Rapid, cost-effective high throughput analysis methods are important for screening large numbers of samples. Simple, cost-effective screening methods using near-infrared spectroscopy (NIR), ultraviolet, and high performance thin layer chromatography (HPTLC) coupled with multivariate analysis, which often do not use expensive instrumentation or extensive trainings, have been commonly used in the agricultural and food industries for classifying foods based on quality traits [26,27]. Multiple screening techniques can be used based on project needs and instrument availability. In our earlier studies, we used ultraviolet-visible (UV-Vis), NIR, nuclear magnetic resonance (NMR), and direct mass spectrometry (MS) for screening food samples grown under different conditions [28,29,30,31].

In the present study, we firstly investigated if rapid screening methods, namely NIR and high-performance thin layer chromatography (HPTLC), coupled with multivariate analysis for rapid classification of 14 soybean samples. Secondly, we analyzed the compositional data of non-polar and polar metabolites such as crude fat content (commonly referred to as oil), fatty acid methyl ester profiles of the transesterified oil, total soluble sugar content, and six sugars profiles in 14 samples (S1–S14) belonging to wild (*Glycine soja*) and cultivated (*Glycine max*) soybeans to identify the components responsible for variation between different soybean samples. Furthermore, correlations between non-polar (total oil and fatty acid methyl esters (FAMEs)) and polar (total soluble sugars content and their profiles) metabolites were also assessed. This study serves as a proof-of-concept to differentiate value-added food and agricultural products from regular commercial products.

## 2. Materials and Methods

### 2.1. Samples

Fourteen soybean genotypes were selected to represent a wide range of genetic diversity. These 14 samples can be clustered into four groups including wild (S3, S12–S14), Asian landraces of *G. max* (S2, S5, S6, and S8), ancestral (S7 and S9), and modern elite (S1, S4, S10, and S11), as shown in Table 1. All soybean samples were obtained from the soybean germplasm collection (USDA, Urbana, IL, USA). All samples were grown in Michigan by Prof. Dechun Wang. The accession number (A#), origin (O), maturity group (MG-0-II), and genotype (wild soybean (*G. soja*) (W), soybean bred for seed traits (SB), soybean landraces (SL), and soybean bred samples (SBS)) information for each sample are provided in Table 1. 

### 2.2. Solvents and Materials

All HPLC grade solvents, chemicals, and HPTLC plates (10 × 20 cm) were purchased from Sigma-Aldrich Chemical Co. (St. Louis, MO, USA). All the individual sugar standards were purchased from Sigma Aldrich (St. Louis, MO, USA) and fatty acid methyl esters standards were purchased from Nu Chek Prep, Inc. (Elysian, MN, USA). Polyvinylidene difluoride (PVDF) syringe filters with a pore size of 0.45 μm were purchased from National Scientific Company (Duluth, GA, USA).

### 2.3. Near Infrared Spectral Fingerprinting

Ground soybean samples were scanned with a near infrared instrument (Thermo Nicolet 6700, (Thermo Scientific, Waltham, MA, USA) spectrophotometer fitted with an InGaAs detector and a CaF_2_ beam splitter. Spectral scans were collected between the regions 4000–10,000 cm^−1^ with 16 cm^−1^ resolution. Each sample was analyzed in triplicate and the glass vials were shaken well and packed by tapping on a soft pad 3–5 times. The spectra were converted to ASCII format and transferred to a PC workstation for statistical analysis.

### 2.4. High-Performance Thin Layer Chromatography (HPTLC)

HPTLC analyses were performed on silica gel 60F254 plates (20 × 10 cm, Merck, Darmstadt, Germany), using a Camag HPTLC system (Camag, Muttenz, Switzerland) equipped with a manual Linomat 5 sampler, twin trough development chamber fitted for 20 × 10 cm plates, and a TLC II visualizer. All modules were controlled with visioncats software (Camag, Muttenz, Switzerland). Standards and sample solutions were applied with a band length of 8 mm at a fixed spray volume of 12 µL per band. Hexane extracts of 14 soybean cultivars were applied on a single silica gel plate. After 30 s of pre-drying, plates were developed at room temperature with a mobile phase of petroleum ether/hexanes (70:30, *v*/*v*) with 0.1% formic acid (10 mL) for a maximum migration distance of 70 mm. The development chamber was vapor saturated with the mobile phase solution before each run. For post-chromatographic derivatization, the plate was immersed into the 10% sulfuric acid solution with the TLC Immersion Device III (Camag) using a vertical speed of 3 cm/s and 1 s immersion time. Plates were then heated in the oven at 100 °C for ~10 min. Dried plates were then visualized in white light. Digital images of the plates were documented by the TLC Visualizer II documentation system equipped with a high-resolution 12 bit CCD digital camera (Camag). Plates were scanned in absorbance normal white light mode, 254, and a 366 nm with deuterium and tungsten lamp slit dimensions of 8.00 mm and 90 mm, respectively; scanning speed of 20 mm/s; and data resolution of 100 lm/step. The data from the scan were converted to csv format for principal component analysis (PCA), which was performed using Solo software.

### 2.5. Analysis of Nonpolar Metabolites (Crude Oil Content and Fatty Acid Methyl Esters Profiles)

The ground soybean seeds were weighed (100 mg) separately and were extracted twice with hexane (5 mL). Each extraction was performed in an ultrasonic bath (power 600 watts) for a period of 15 min. The combined extracts were centrifuged at 5000 rpm for 10 min and the supernatant hexane layer was collected. The extracts were filtered through a 0.45 µm PVDF syringe filter into a pre-weighed 15 mL tube and the hexane extracts were evaporated to dryness under a slow stream of nitrogen gas. The tubes were reweighed to determine the percent oil extracted from ground soybeans. The oil was re-suspended with 2 mL hexane and, from that, 1 mL was separated and evaporated to dryness for the preparation of fatty acid methyl ester derivatives by transesterification of extracted soybean oil. The second aliquot of the oil extract was assayed by HPTLC analysis.

For FAMEs, derivatization was performed using 5 mL of acidified methanol (10 mL of acetyl chloride to 90 mL of cold methanol). The mixture was kept at ambient temperature overnight with continuous stirring, and then 3 mL of water was added. The fatty acid methyl esters were extracted with 2 mL of hexane. The hexane layer containing FAMEs was separated and analyzed with Agilent GC 6890 (Agilent Technologies, Santa Clara, CA, USA) attached to a flame ionization detector [32]. For FAMEs’ separation and analysis, the oven temperature was set at 170 °C with an equilibration time of 3 min. After 3 min, the temperature was gradually increased to 215 °C with a ramp of 10 °C. Helium was used as a carrier gas. The column used was Omegawax^TM^ 250 (30 m × 0.25 mm × 0.25 µm, Sigma Aldrich, St. Louis, MO, USA)

### 2.6. Analysis of Polar Metabolites (Soluble Sugar Content and Profiles)

The soluble sugars were extracted as reported previously [33] using ultrasonic-assisted extraction. For each analysis, 25 mg of ground soybean sample was extracted with 5 mL of deionized (DI) water (4 mL of DI water + 1 mL DI water containing xylitol, 5 mg/mL). The mixture was sonicated in the ultrasonic bath for 1 h. Samples were then centrifuged at 4000 rpm for 10 min. The supernatant was decanted into a filtration syringe fitted with a 0.45 μm PVDF syringe filter. The filtered extracts were assayed by ion chromatography coupled to an electrochemical detector. All extracted were further diluted 100 times prior to analysis. Initially, a method was developed and validated for the separation and quantification of six sugars (glucose, galactose, fructose, sucrose, raffinose, and stachyose) and xylitol (internal standard), using a Dionex ICS-5000 IC system (Dionex, Thermo Fisher, Sunnyvale, CA, USA). The system consisted of a Dionex gradient pump and electrochemical detector equipped with an amperometric cell. The cell consisted of a 1.0 mm diameter gold working electrode. pH; Ag/AgCl was used as the reference electrode. The separations were carried out on a CarboPac PA 20 column set consisting of a guard column (3 × 30 mm) and an analytical column (3 × 150 mm). The sample injection volume was 100 μL. The column was placed inside a temperature controller, which was maintained at 50 °C. The flow rate was 0.4 mL min^−1^ and the total chromatography runtime was 15 min. The data acquisition and analysis were performed using Chromeleon software 6.8 (Dionex, Thermo Fisher, Sunnyvale, CA, USA). Calibration curves with individual sugar standards were plotted using a log scale. Linearity range, precision, limit of detection (LOD), and limit of quantitation (LOQ) for individual sugars were determined as per International Council for Harmonization (ICH) guidelines [34]. Total sugars were computed by adding the amount of six identified soluble sugars. The molecular weights for the sugars were confirmed with a single quadrupole mass spectrometer (Thermo Scientific MSQ Plus, Waltham, MA, USA) instrument [33]. Mass spectra were obtained using electrospray ionization in positive mode within a mass range of 125–800 m/z. Mass spectrometry conditions were as follows: needle voltage, 3000 V; cone voltage, 80 V; probe temperature, 450 °C; nitrogen (drying gas) pressure, 80 psi.

### 2.7. Statistical Analysis

All NIR spectral data were exported from the spectrometers to Excel (Microsoft, Inc., Belleview, WA, USA) for pre-processing. Hierarchical cluster analysis and PCA were performed using Solo (Eigenvector Research, Inc., Wenatchee, WA, USA). Correlations between total crude fat and total soluble sugar content along with the individual FAMEs and sugar were calculated using the COR function in R. A heatmap was generated in R using ggheatmap.

## 3. Results and Discussion

**Figure 1:**
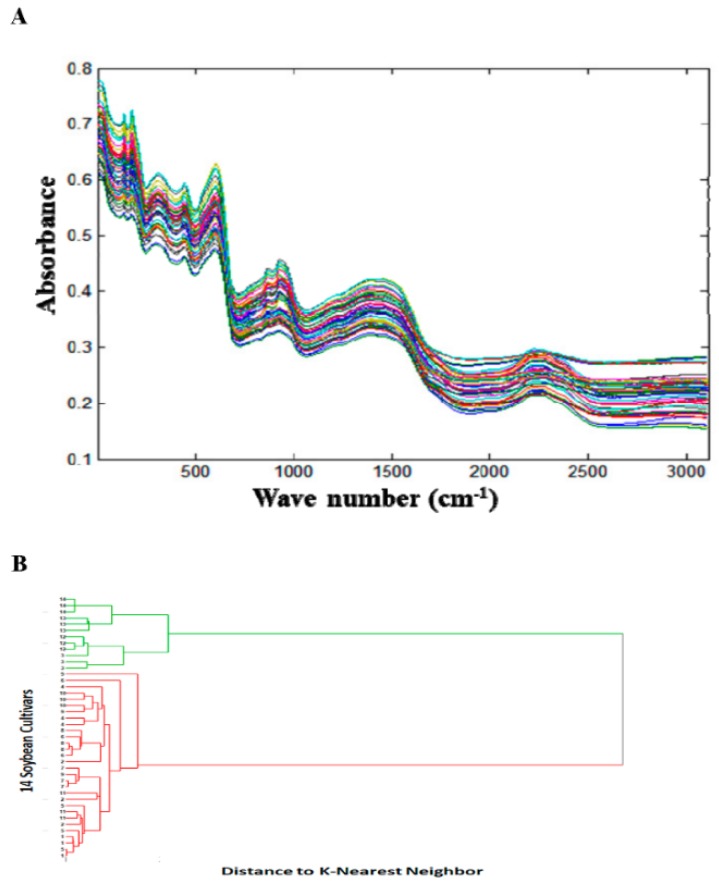
(**A**) Near infrared spectroscopy (NIR) spectral fingerprints of 14 ground soybean samples and (**B**) hierarchical cluster analysis of the near infrared spectral data of 14 soybeans samples. All spectral data were collected in triplicates.

The NIR spectral fingerprints were collected to evaluate qualitative differences among 14 soybean cultivars (Figure 1A). The hierarchical cluster analysis of the first derivative of the NIR spectral absorption data followed by normalization and distance to K-nearest preprocessing method showed two distinct clusters, one for the wild soybeans (*G. soja*, S3, S12–S14) and another for cultivated soybeans (*G. max*, S1, S2, S4–S11) for all three replicate spectral fingerprints (Figure 1B). Similar results were obtained with PCA analysis of the first derivative of the NIR spectral absorption data followed by normalization for all three replicates. These results suggested that there were significant compositional differences between wild and cultivated soybeans.

**Figure 2:**
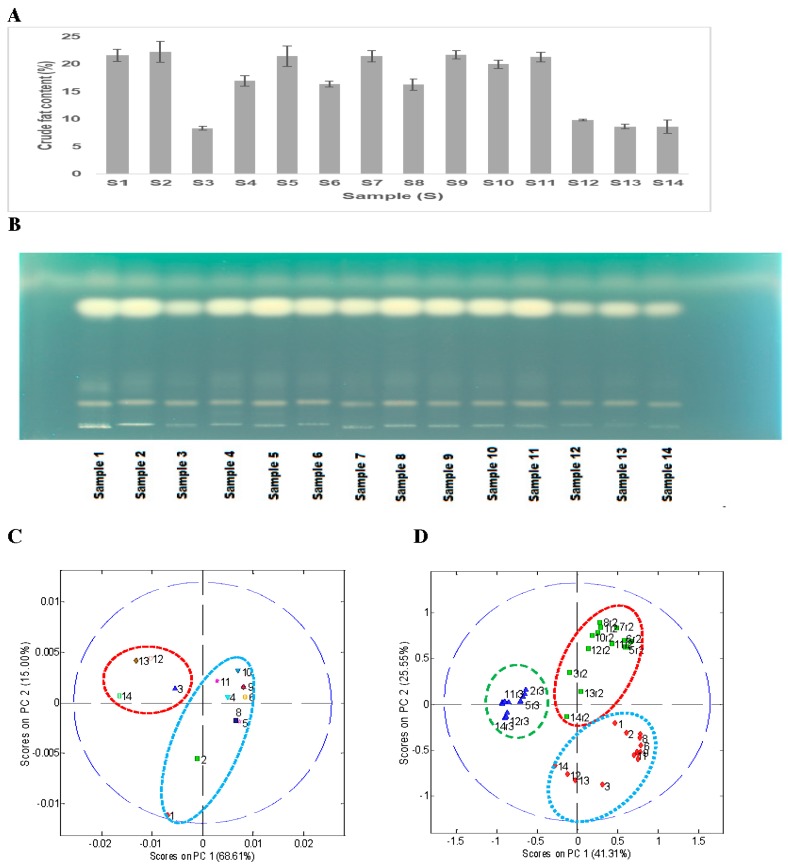
(**A**) Total crude fat content, (**B**) high-performance thin layer chromatography (HPTLC) analysis of the crude fat hexane extract form 14 soybean samples, (**C**) a typical principal component analysis (PCA) analysis score plot of hexane extract HPTLC data from a single repetition, and (**D**) PCA analysis score plot of the HPTLC data of 14 soybean hexane extracts from all three repetitions (r1 red, r2 green, and r3 blue) analyzed on separate HPTLC plates.

Soybeans were extracted with hexane and water separately to evaluate if the above variations were because of nonpolar or polar metabolites. Figure 2A shows the total crude fat content of the 14 soybean samples. The total crude fat (oil) content ranged from 8.3% to 22.3%. All wild type soybean samples (S3, S12–S14) have a lower total crude fat content (8.9%) than the cultivated soybeans (S1, S2, S4–11), which contained around 20% crude fat content. A similar low yield of total oil content (~11%) in wild soybeans (*G. soja*) was reported in a recent study by Leamy et al. (2017) [35]. The average crude fat content in the cultivated soybeans (*G. max*) varied between 16.3% and 22.3%, which was similar to the range (15.9% to 19.5%) reported in the literature [36].

This was followed by HPTLC analysis of the hexane extract to see if there was any significant variation in different types of nonpolar metabolites (free fatty acids, sterols, sterol esters, triglycerides, diacylglycerols, monoacylglycerols, free fatty acid methyl esters, or other non-polar compounds). A simple HPTLC analysis of the hexane extract of the 14 soybean samples showed a significant difference in the oil (triglycerides reference) content in the wild (*G. soja*, S3, S12–S14) and cultivated soybeans (*G. max*, S1, S2, S4–11) (Figure 2B). The top band of triglyceride at Rf (~0.73) showed lower band intensity for wild type samples (S3, S12–14) than cultivated ones (S1, S2, S4–11).

Principal component analysis of the peak areas from the HPTLC results after alignment showed two distinct clusters as expected, one for the wild type soybeans (*G soja*, S3, S12–S14) with low oil content as compared with the other for the cultivated soybeans (*G. max*, S1, S2, S4–S11) (Figure 2C). However, PCA analysis of all three replicates of the HPTLC datasets based on Rf only showed the presence of three separate clusters based on the repetition (Figure 2D). All 14 soybean hexane extract samples from plate 1 clustered together, and similar results were observed with plate 2 and plate 3 (Figure 2D). As part of the HPTLC analysis was performed in a manual way, the Rf of the major triglyceride band on the three plates was marginally different (plate 1, Rf = 0.74; plate 2, Rf = 0.77; and plate 3, Rf = 0.87), which caused separate clustering for each of the three-replicate analyses.

**Figure 3:**
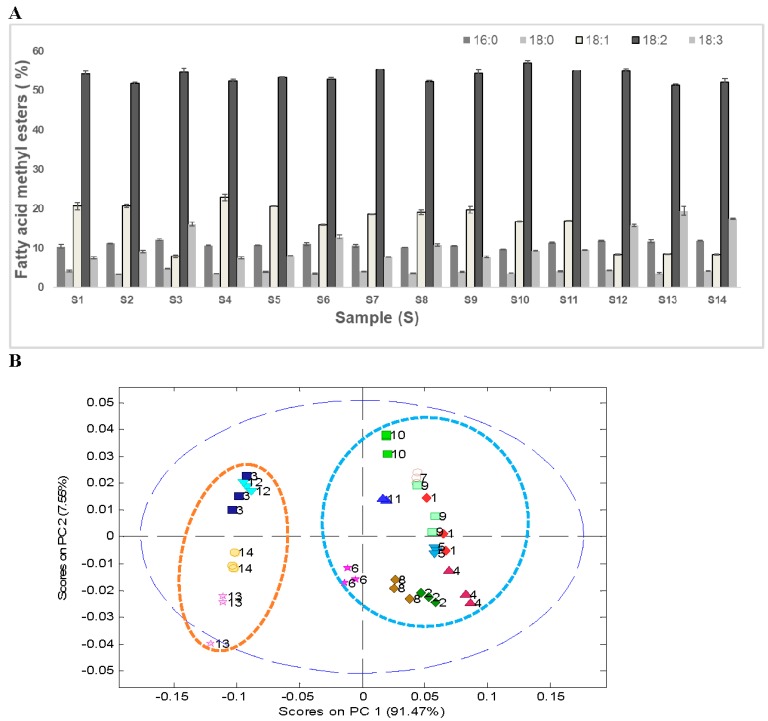
(**A**) Fatty acid methyl ester composition of the transesterified oil from 14 soybean cultivars and (**B**) principal component analyses of the fatty acid methyl ester composition data. All extractions and analyses were performed in triplicate and the error bar represents standard deviation between three or four replicate analyses.

Gas chromatography analysis after transesterification of the oil extracted with hexane was used to identify variations in the FAMEs’ composition. The results of GC analysis showed the presence of five prominent FAMEs, namely, methyl palmitate, methyl stearate, methyl oleate, methyl linoleate, and methyl linolenate, which accounted for around 95% of the total FAMEs’ area. The FAMEs’ composition of the 14 soybean samples is presented in Figure 3A. The predominant FAME in all soybean samples was methyl linoleate, which accounted for about 51%–57%. The average saturated FAME content in 14 soybean samples was around 15%. Only a marginal difference (~2%) in the saturated fatty acid composition was observed in wild soybeans (~16%) compared with the cultivated soybeans (~14%). Both methyl oleate and methyl linolenate varied significantly between the ranges 8%–23% and 8%–19%, respectively, in 14 soybean samples.

PCA analysis of the FAMEs’ data showed a distinct clustering pattern between wild soybeans (S3, S12–S14) and cultivated soybeans (S1, S2, S4–S11, Figure 3B). These differences can primarily be attributed to the lower quantity of methyl oleate (~8%) and higher quantity of methyl linolenate (~17%) in wild soybeans (*G. soja*, S3, S12–S14). On the comparative basis, a significantly higher quantity of methyl oleate (~19%) and lower quantity of methyl linolenate (~9%) was observed in cultivated soybeans (*G. max*, S1, S2, S4–S11). Similar FAMEs’ composition of the transesterified soybean oil has been reported in the literature, where authors showed low levels of oleic acid (~7.5%–15.8%) in wild soybeans from Japan as compared with commercial varieties, which contained 20%–25% oleic acid [37]. The same group of authors reported comparatively higher levels (19%) of linolenic acids in wild type soybeans as compared with commercial soybeans, where the linolenic acids levels were around 8%–10% [37].

The polar metabolites (soluble sugars) from the water extracts, were assayed by an IC-ECD method. This procedure was developed and validated to quantify the soluble sugars in extracted soybean samples for linearity, precision, LOQ, and LOD. MSD was used to confirm the masses (m/z values) of sugars extracted from soybean samples, as reported previously [33]. Calibration curves were plotted for the log area ratio of analyte (sugar) to IS versus concentration. For this purpose, seven concentrations (0.1 µg/mL, 0.2 µg/mL, 0.5 µg/mL, 1.0 µg/mL, 2.0 µg/mL, 5.0 µg/mL, and 10 µg/mL) of each sugar and 10 µg/mL of xylitol was used as an IS. Precision was measured as relative standard deviation (RSD) using six replicates of the peak area ratio of each sugar to IS for three different concentrations (0.5 µg/mL, 2 µg/mL, and 10 µg/mL). LOD and LOQ were calculated for six replicates of single standard as 3.3/10 times the standard deviation divided by the slope of the calibration curve for each sugar [24,34,38]. The analytical method data for quantification of six sugars is shown in Table 2. All sugars showed good linearity with a minimum of *R*^2^ = 0.99 with the linearity range of 0.1–10 µg/mL. The LOQ range was found to be 0.010–0.048 µg/mL, whereas the LOD range was found to be 0.003–0.016 µg/mL for the six sugars.

**Figure 4:**
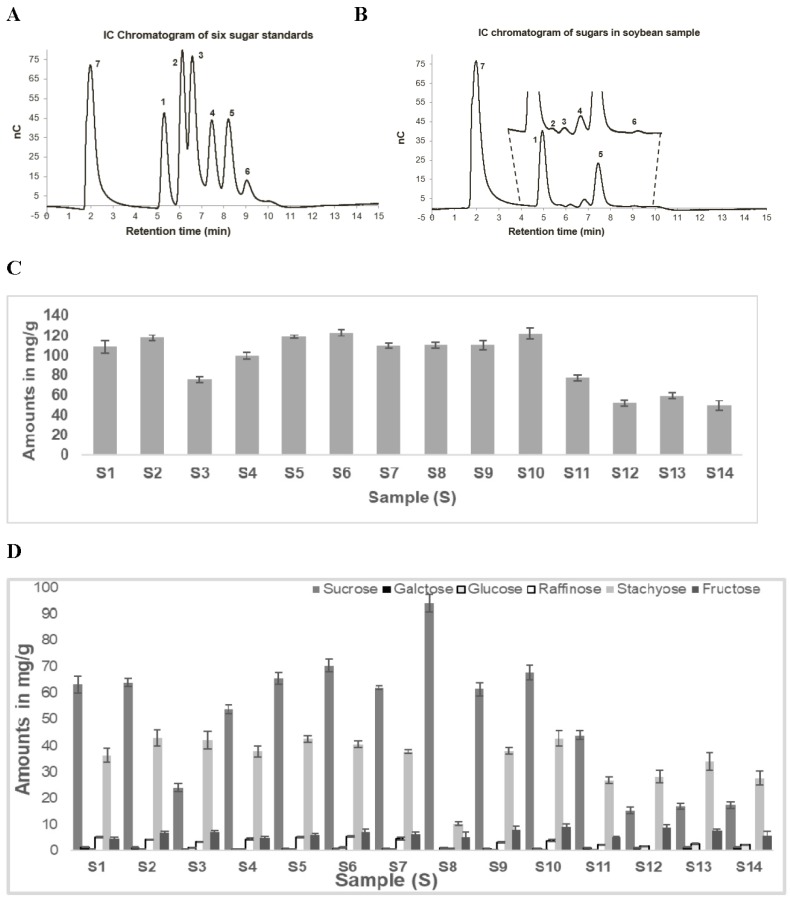
A representative chromatogram depicting the separation of sugars (Sucrose **1**, Galactose **2**, Glucose **3**, Raffinose **4**, Stachyose **5**, Fructose **6**) and the internal standard (Xylitol **7**). (**A**) Six sugar standards, (**B**) sugars extracted from a soybean sample, (**C**) total soluble sugar content, and (**D**) individual soluble sugar profiles of 14 soybean samples. All extractions and analyses were performed in triplicate and the error bar represents standard deviation between three or four replicate analyses. IC, ion chromatography.

Figure 4A shows the representative chromatogram for the separation of six standards (sucrose-retention time (RT) 5.2 min, galactose-RT 6.1 min, glucose-RT 6.5 min, raffinose-RT 7.4 min, stachyose-RT 8.2 min, and fructose-RT 9.2 min) for sugars commonly detected in soybean samples along with xylitol (RT 2.0 min), the internal standard (IS). The total runtime for the separation of six sugars along with xylitol was 15 min and all seven analytes were eluted within 10 min. One of the main advantages of the IC-ECD method is high sensitivity. Some of the sugars (fructose, glucose, and galactose) found in soybeans are in low concentrations, hence sensitive analytical methods for quantification are desirable for the quantification.

A typical chromatogram of soluble sugars extracted from a soybean sample is presented in Figure 4B. The total soluble sugar content for all 14 soybean samples is presented in Figure 4C. Triplicate analyses of all three-biological replicates are reported. Thus, each sample was analyzed nine times. A wide variation (51.4 mg/g to 123.7 mg/g) in the total soluble sugar content was observed among 14 soybean samples. The average total soluble sugars in wild soybeans (59.8 mg/g) were approximately half the average of cultivated soybeans (110.0 mg/g). A similar difference in sugar content in wild and cultivated soybeans was observed by Hou et al. (2009). Sucrose and stachyose were found to be the two predominant sugars in all soybean samples (79%–94%). The concentration of the two predominant sugars in cultivated soybeans (S1, S2, S4–11) varied between 88% and 94% as compared with marginally lower amounts (79%–84%) in wild type soybeans (S3, S12–S14). Moreover, the amounts of sucrose and stachyose in these samples varied based on the type of soybeans studied (Figure 4D). For all wild type samples, stachyose was the major sugar and varied between 51% and 55%, while the concentration of sucrose was 27%–32%. The average sucrose and stachyose percentage in the cultivated soybean samples was determined as 59% and 32% respectively. This was interchanged for the wild type soybeans. Interestingly, sample 8 had a unique sugar profile, with sucrose constituting around 86% of the total sugar and stachyose content being only about 9%. The abundance of the other sugars was significantly lower than the other two sugars and varied between 5% and 20%. Similar sugar profiles for wild and cultivated soybeans have been described in the recent literature [13]. The differences between the wild and cultivated soybean were primarily because of the proportion of the two prominent sugars, stachyose and sucrose content, as described earlier.

**Figure 5:**
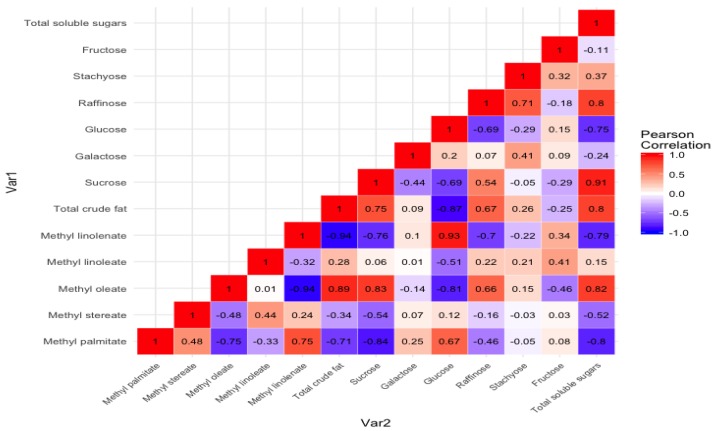
Heatmap showing correlation between total crude fat and total soluble sugar content along with the individual FAMEs and sugar profiles. FAMEs: fatty acid methyl esters.

Figure 5 shows the Pearson correlation between the nonpolar (oil content and their FAMEs’ profile) and polar (soluble sugar content and their profile). In addition, the correlation between individual sugars and fatty acid methyl esters profile is also depicted. The results showed a positive correlation (*R*^2^ = 0.80) between the total crude fat content and the total soluble sugar content. A similar, but weak positive correlation (*R*^2^ = 0.36) between oil and carbohydrate content was observed by Hymowitz et al. (1972) while studying composition in 60 soybean lines. A strong positive correlation between total soluble sugar and sucrose (*R*^2^ = 0.91) and total soluble sugar and raffinose was observed (*R*^2^ = 0.80). Similar strong correlations between total sugar and sucrose (*R*^2^ = 0.82) and total soluble sugar and raffinose (*R*^2^ = 0.64) were observed in the worldwide collection of 241 soybean germplasm by Hou et al. 2009. However, the same group also showed strong correlation between glucose and fructose (*R*^2^ = 0.98), which was insignificant (*R*^2^ = 0.15) in the present study.

Positive correlation between sucrose (*R*^2^ = 0.83), total sugar content (*R*^2^ = 0.82), and methyl oleate was observed. Furthermore, a strong negative correlation between methyl linolenate and total soluble sugar content (*R*^2^ = −0.79), total crude fat content (*R*^2^ = −0.94), and monounsaturated fatty acid methyl ester (methyl oleate) (*R*^2^ = −0.94) was observed in the present study. This information may provide insights for breeders and biotech professionals to develop better quality soybeans with improved oil and soluble sugar profiles.

## 4. Conclusions

In the present study, we used NIR, HPTLC, and multivariate analysis techniques for rapid qualitative classification of 14 soybeans, which showed distinct differences between wild type and cultivated soybeans. We determined and compared the oil content, soluble sugar content, FAMEs, and soluble sugar profiles in four wild (*Glycine soja*) and ten cultivated soybeans (*Glycine max*) by GC-FID and IC-ECD-MS methods. Multivariate analysis of HPTLC, NIR, total oil and sugar content, FAMEs, and the sugar profiles data analysis also showed a distinct separation between the wild and cultivated soybean samples. These variations were attributed to wide differences in the oil and sugar content, the oil content in wild soybean was 9% as compared to 16%–22% in cultivated soybeans. Similarly, a higher level of linolenic acid (~17%) and lower oleic acid (~9%) were observed in wild type soybeans as compared with lower linolenic acid (~9%) and higher oleic acid (~19%) in cultivated soybeans. The sugar profiles in wild type soybeans contained higher levels of stachyose as compared with higher levels of sucrose in cultivated soybeans. Cultivated sample 8 had a unique sugar profile with very high amounts of sucrose (86%) and very low abundance of not easily digestible sugar stachyose (9%). Positive correlations between total oil content and total soluble sugar content (*R*^2^ = 0.80) and negative correlations between methyl linolenate and total soluble sugar content (*R*^2^ = −0.79), total crude fat content (*R*^2^ = −0.94), and monounsaturated fatty acid methyl ester (methyl oleate) (*R*^2^ = −0.94) were observed.

## Figures and Tables

**Table 1:** Details of the 14 soybean samples used for the current study.

Accession Number	Origin/Information	Maturity Group	Sample	Genotypes
PI 614088	Illinois, USA	II	Sample 1	Soybean Bred for Seed Traits
Mandarin (Ottawa) PI 548379	Selected from ‘Mandarin’ in 1929	0	Sample 2	Soybean Landraces
PI 597448D	China	0	Sample 3	Wild soybean (*G. soja*)
PI 07158T	Michigan, USA	II	Sample 4	Soybean Bred for Seed Traits
Richland PI 548406	Selected from PI 70502 in 1927	II	Sample 5	Soybean Landraces
PI 416757	Japan	I	Sample 6	Soybean Landraces
Parker PI 562374	Minnesota, USA	I	Sample 7	Soybean Bred for seed Traits
PI 200508	Japan	I	Sample 8	Soybean Landraces
Surge PI 599300	South Dakota, USA	0	Sample 9	Soybean Bred for seed Traits
PI 533655	Illinois, USA	II	Sample 10	Soybean Bred for Seed Traits
PI 597386	Illinois, USA	II	Sample 11	Soybean Bred for Seed Traits
PI 424004B	South Korea	II	Sample 12	Wild soybean (*G. soja*)
PI 464890B	China	I	Sample 13	Wild soybean (*G. soja*)
PI 342622A	Russia	I	Sample 14	Wild soybean (*G. soja*)

PI denotes plant introduction.

**Table 2:** Analytical method assessment for ionic chromatography of six sugars. LOQ, limit of quantitation; LOD, limit of detection.

Sugar	Linearity (µg/mL)	Regression Equation	*R* ^2^	LOQ (µg/mL)	LOD (µg/mL)	RSD%					
						Intra (*n* = 3)			Inter (*n* = 6; 2 × 3)		
						Low	Medium	High	Low	Medium	High
Sucrose	0.1–10	y = 0.9764x − 3.9599	0.9976	0.025	0.008	0.41	0.99	1.64	2.62	1.95	1.19
Galactose	0.1–10	y = 0.9659x − 3.7761	0.9991	0.040	0.013	4.04	1.59	2.30	4.01	3.74	1.63
Glucose	0.1–10	y = 0.9645x − 3.6356	0.9948	0.044	0.015	0.40	1.64	1.10	0.84	2.35	2.09
Raffinose	0.1–10	y = 0.9861x − 3.9454	0.9945	0.038	0.013	1.89	1.77	2.23	3.58	3.08	4.40
Stachyose	0.1–10	y = 1.0434x − 4.1417	0.9941	0.048	0.016	2.31	2.19	3.35	4.40	3.35	4.18
Fructose	0.1–10	y = 1.1205x − 5.0196	0.9918	0.010	0.003	2.84	4.84	3.21	5.50	8.82	11.00
